# Genome Sequence of *Serratia marcescens* subsp. *sakuensis* Strain K27, a Marine Bacterium Isolated from Sponge (*Haliclona amboinensis*)

**DOI:** 10.1128/genomeA.00022-18

**Published:** 2018-02-08

**Authors:** Tzi Him Cheng, Jasnizat Saidin, Muhd Danish-Daniel, Han Ming Gan, Mohd Noor Mat Isa, Mohd Faizal Abu Bakar, Noraznawati Ismail

**Affiliations:** a Institute of Marine Biotechnology, Universiti Malaysia Terengganu, Kuala Terengganu, Terengganu, Malaysia; b School of Life and Environmental Sciences, Deakin University, Geelong Waurn Ponds, Australia; c Malaysia Genome Institute, Jalan Bangi, Kajang, Selangor, Malaysia

## Abstract

*Serratia marcescens* subsp. *sakuensis* strain K27 was isolated from sponge (*Haliclona amboinensis*). The genome of this strain consists of 5,325,727 bp, with 5,140 open reading frames (ORFs), 3 rRNAs, and 67 tRNAs. It contains genes for the production of amylases, lipases, and proteases. Gene clusters for the biosynthesis of nonribosomal peptides and thiopeptide were also identified.

## GENOME ANNOUNCEMENT

*Serratia marcescens* subsp. *sakuensis* was first isolated from a domestic wastewater tank in 2003. It is the only *Serratia* species that produced spores, and it has only minor differences from *Serratia marcescens* in terms of its biochemical test (methyl red test and production of acid from l-arabitol) (1). *Serratia marcescens*, a Gram-negative *Enterobacteriaceae* organism, became an important cause of nosocomial infection in the 1960s (2). *Serratia marcescens* subsp. *sakuensis* strain K27 was isolated from sponge (*Haliclona amboinensis*). The genome of this strain was sequenced in order to gain insights into the genes involved in the biosynthesis and production of enzymes useful in industrial applications.

*S. marcescens* subsp. *sakuensis* strain K27 was cultured in Marine Broth 2216 (Difco). The genomic DNA was then extracted using the Qiagen DNeasy tissue kit (Qiagen, Inc., Valencia, CA, USA). Sequencing was performed on the Illumina MiSeq platform, generating 294,464,639 raw FASTQ paired-end reads, and was *de novo* assembled using Velvet version 1.2.09 (3). The total sequence length was 5,325,727 bp, with 55× genome coverage, and the *N*_50_ was determined to be 67,510 bp.

Prodigal version 2.60, RNAmmer version 1.2, and tRNAscan-SE version 1.3.1 were used to annotate the genome, predicting 5,140 open reading frames, 10 rRNAs, and 67 tRNAs, respectively (4–6). The predicted 16S rRNA was queried with BLASTn (7) against the nucleotide collection database and confirmed that the strain was *S. marcescens* subsp. *sakuensis* with 99% similarity to *S. marcescens* subsp. *sakuensis* in GenBank (accession number NR_036886). Further validation of species and subspecies was confirmed with the EzBioCloud database, which showed that strain K27 is most similar to *S. marcescens* subsp. *sakuensis* KCTC 42172 (CLG_48654), with a probability of 100% (8). Protein functions were predicted using InterProScan5 (9), while the presence of a secondary metabolite biosynthesis gene cluster in the genome was identified by antiSMASH (10).

To date, no study has reported on the genome sequence of this subspecies. Here, we report the first draft genome sequence of *S. marcescens* subsp. *sakuensis*. Analysis of the genome revealed industrial enzyme-related genes for amylases (contig 204), lipases (contigs 48, 74, 107, 113, 126, 128, and 270), and proteases (contigs 24, 59, 64, 104, 132, 138, 167, 176, 180, 182, 187, 192, 254, 355, and 356). Also, genes for environment tolerance (contig 167) and thermal tolerance (contigs 11 and 182) and a cold-shock protein-coding gene (contigs 102, 105, 172, 187, 221, and 260) were found in this genome, indicating that this strain can produce enzyme(s) with environmental stress tolerance, especially for low temperature. Furthermore, several gene clusters for the biosynthesis of nonribosomal peptides and thiopeptide have been identified (contigs 10, 16, 88, 126, 180, 187, and 260). This suggests that the genome of this strain is important for further study and may contain novel genes other than previously reported genes found in *Serratia marcescens*.

### Accession number(s).

This whole-genome shotgun project has been deposited at DDBJ/ENA/GenBank under the accession number PESM00000000. The version described in this paper is version PESM01000000.
